# Hybrid Photonic Cavity with Metal-Organic Framework Coatings for the Ultra-Sensitive Detection of Volatile Organic Compounds with High Immunity to Humidity

**DOI:** 10.1038/srep41640

**Published:** 2017-01-31

**Authors:** Jifang Tao, Xuerui Wang, Tao Sun, Hong Cai, Yuxiang Wang, Tong Lin, Dongliang Fu, Lennon Lee Yao Ting, Yuandong Gu, Dan Zhao

**Affiliations:** 1 Institute of Microelectronics, A*STAR (Agency for Science, Technology and Research), 2 Fusionopolis Way, #08-02 Innovis Tower, 138634, Singapore; 2 Department of Chemical & Biomolecular Engineering, National University of Singapore, 4 Engineering Drive 4, 117585, Singapore

## Abstract

Detection of volatile organic compounds (VOCs) at parts-per-billion (ppb) level is one of the most challenging tasks for miniature gas sensors because of the high requirement on sensitivity and the possible interference from moisture. Herein, for the first time, we present a novel platform based on a hybrid photonic cavity with metal-organic framework (MOF) coatings for VOCs detection. We have fabricated a compact gas sensor with detection limitation ranging from 29 to 99 ppb for various VOCs including styrene, toluene, benzene, propylene and methanol. Compared to the photonic cavity without coating, the MOF-coated solution exhibits a sensitivity enhancement factor up to 1000. The present results have demonstrated great potential of MOF-coated photonic resonators in miniaturized gas sensing applications.

Volatile organic compounds (VOCs), such as xylene, benzene, and formaldehyde, are those chemicals with high vapor pressures at room temperature. They exist extensively in our daily life and may cause health problems including cancer, headaches, central nervous system damage, *etc.*^1^,^2^,^3^,^4^,^5^,^6^,^7^. It is therefore of great importance to develop sensitive and reliable detection technologies for VOCs to monitor their health hazard. Besides health issues, VOCs detection is also essential in several other applications such as food safety^8^, cosmetic industry^9^, and diagnosis of diseases as biomarkers through breath analysis^10^. Currently, gas-phase VOCs detection is mainly conducted by gas chromatograph (GC) or mass spectrometer (MS), whose bulky size and high cost limit their wider applications^11^,^12^. Some other compact gas-phase VOCs sensors based on nanocantilevers^13^, graphane^14^,^15^ or carbon nano-tubes^16^,^17^ were demonstrated with very high sensitivity and fast response, but their sensitivity drifts seriously in long time running and they are unstable to be used in real test conditions due to strong cross-response to humidity^18^. Another alternative technology for VOCs sensing is absorption spectroscopy, which measures the optical power variation in photon-gas interaction and have unique advantages such as low cross-response, fast response and high accuracy^19^,^20^. However, the absorption spectra of VOCs normally cover a broad range from ultraviolet to middle infrared, and the absorption coefficients are relative weak^21^,^22^,^23^,^24^. It is very difficult to realize ppb-level detection. Up to now, to build a universal optical sensing platform for VOCs detection is still quite challenging.

Photonic micro-ring resonator (MRR) is a powerful detection and analysis tool that has vast applications in biomedical research^25^,^26^, environmental monitoring^27^ and pharmaceuticals^28^. In a MRR, the light propagates in the form of circulating waveguide modes (CWMs) coming from total internal reflection of the curved boundary between the high and low refractive index (RI) medium^29^. The CWMs generate evanescent fields at outside of the MRR and respond to changes in the dielectric properties^30^ of the surroundings. To quantify the sensing capability, the detection limit (DL) with a unit of refractive index unit (RIU) is used (typically in the range of 10^−7^~10^−5^), which is the ratio of the resonance shift of the resonator to the local refractive index of the medium. DL can be improved by increasing the light-matter interactions, such as to pre-concentrate analytes onto resonators^31^. Currently, most of the reported materials used for pre-concentration are polymers, such as polyimide and polydimethylsiloxane^29^. They are easy to form coating layers but are limited by long-term stability and poor gas adsorption capability. Metal-organic frameworks (MOFs) are emerging porous materials composed of organic ligands and inorganic metal clusters^32^,^33^,^34^,^35^,^36^. They have the unique advantages of large specific surface areas and mechanical stability close to that of crystalline semiconductors^40^. Moreover, their growth and patterning by standard semiconductor fabrication processes have been demonstrated recently^36^,^37^,^38^,^39^. Herein, we report the fabrication of MRR coated with MOF layers serving as pre-concentrating materials for enhanced detection sensitivity toward gas-phase VOCs.

## Design and Fabrication

**Figure 1:**
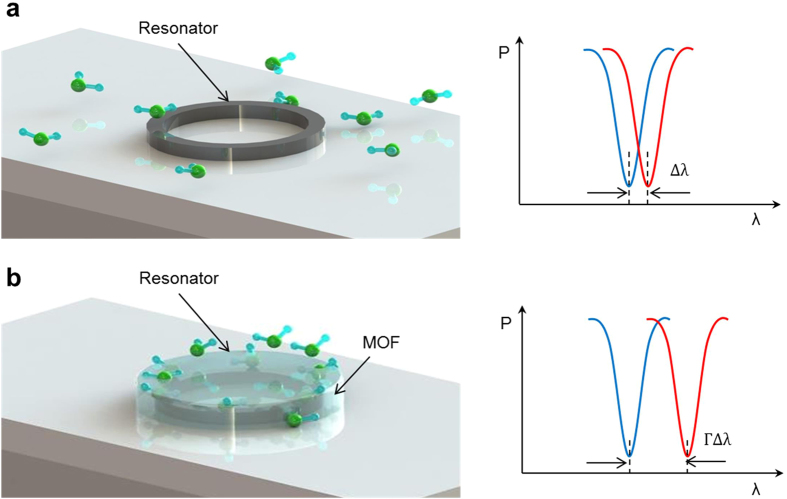
Schematic representation the gas sensing by use of a optical micro-ring resonator. Blue line and red line in spectrum show resonant wavelengths without/with gas. Gas molecules surrounding the micro-ring resonator changes its complex dielectric function, which causes a resonance shift of the micro-ring resonator that can be detected. (**a**) Gas sensing with a normal micro-ring resonator without coating. Spectra are almost overlapped with a barely detectable change (Δλ). (**b**) Gas sensing with the hybrid photonic-MOF device. The spectrum shift induced by gas absorption can be identified clearly (ΓΔλ).

This design fully utilizes the strong absorption properties of MOFs^40^,^41^,^42^ and the ultra-high quality factor (Q-factor) of photonic resonator structures to demonstrate an on-chip VOCs sensing approach with an enhancement of detection factor as high as 10^3^ compared with non-coated ones. Figure 1 illustrates the working mechanism of our approach. The resonant spectra of micro-ring are labeled as blue (blank) and red (under test), respectively. The micro-ring alone causes a barely detectable change (Δλ) upon gas exposure because of the limited amount of gas molecules nearby (Fig. 1a). In contrast, dramatically increased amount of gas molecules can be adsorbed by the MOF coating of the waveguides, leading to an obvious resonance shift (ΓΔλ) of the micro-ring that can be easily detected with increased detection sensitivity (Fig. 1b).

**Figure 2:**
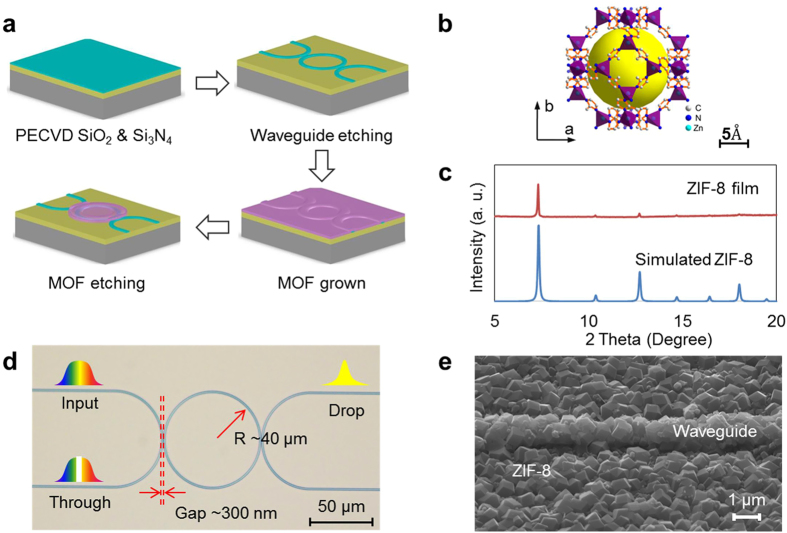
(**a**) Illustration of the fabrication process flow for hybrid photonic-MOF sensor device. (**b**) Chemical structure of the ZIF-8 nanocrystals. (**c**) X-ray diffraction pattern. (**d**) Micrograph of the hybrid photonic-MOF gas sensor. The micro-ring resonator is coupled with two bus waveguides that have two integrated inversed-tip couplers for input and output of optical signals from and to optical fibers. (**e**) Scanning electron microscope image of the top view.

We selected ZIF-8 as the external MOF coating material because of its high surface area (1840 m^2^ g^−1^), good water stability, and high light transmittance in NIR^43^,^44^. ZIF-8 has large cavities (11.6 Å) and small apertures (3.4 Å) that can be expressed as a space-filling packing of regular truncated octahedral, as shown in Fig. 2b. The VOCs molecule can penetrate and are adsorbed within ZIF-8 cavities. The device fabrication processes are shown in Fig. 2a. Silicon nitride (Si_3_N_4_) waveguide was used to fabricate the MRR. It has a radius of 40 μm, and the coupling gap between the bus waveguide and the MRR is around 300 nm. A 23,000 Q-factor is obtained in the testing. Compared with silicon waveguides, Si_3_N_4_ waveguides has a low refractive index that allows a smaller confinement factor inducing a larger evanescent optical field, which results in a strong interaction between the incident light and gas molecules. Fabrication started with a 3.5 μm thick silicon dioxide (SiO_2_) layer and a 400 nm thick Si_3_N_4_ layer deposition in a low-pressure chemical vapour deposition (LPCVD) furnace. Followed by waveguide structures defined using deep UV photolithography, reactive ion etching (RIE) was used to transfer waveguide patterns onto the Si_3_N_4_ layer. After that, a 200 nm thick SiO_2_ cladding layer was blanket deposited on the entire device by plasma-enhanced chemical vapour deposition (PECVD) to reduce the waveguide transmission loss and protect the Si_3_N_4_ waveguides. Then, the photonic device was washed in sulfuric acid before ZIF-8 growth. Layer-by-layer intergrowth process was employed for the synthesis of ZIF-8 coating, whose structure can be confirmed by the powder X-ray diffraction patterns (Fig. 2c). Finally, ZIF-8 thin film was patterned using lithography and etched by sulfuric acid (Fig. 2d). The MRR is side coupled with two bus waveguides to readout the drop and through signals. The morphology of the ZIF-8 coating was inspected by scanning electron microscopes (SEM) as shown in Fig. 2e. The bus waveguides and MRR has a dimension of 1 μm × 0.4 μm, and the thickness of the ZIF-8 coating is around 1 μm. To active the ZIF-8 film, the device was immersed in anhydrous methanol for 3 days with frequently refreshing of methanol to remove the unreacted zinc ions and 2-methylimidazole. After the removal of methanol, the film was dried under a dynamic vacuum at 120 °C for 12 h.

## Experimental Results

**Figure 3:**
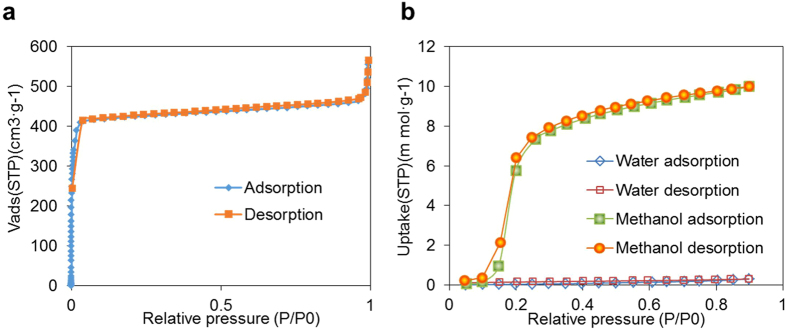
(**a**) N_2_ sorption isotherms at 77 K of ZIF-8. (**b**) Absorption isotherms of water and methanol by ZIF-8 that dedicates the merits of high immunity to humidity, while cross-responding to humidity is regarded as one of most challenges for gas sensors.

To evaluate the performance of the ZIF-8 film on gas adsorption, a gas adsorption experiment has been carried out by use of surface area and porosimetry system (Model: Micromeritics ASAP 2020). Figure 3a shows nitrogen (N_2_) sorption isotherm at 77 K, which exhibits a typical type I isotherm pattern indicating the microporous nature of ZIF-8. The hysteresis loop at high relative pressure shows the existence of mesopores in addition to the microporous network^45^. ZIF-8 is a well-known material with high hydrophobicity adsorbing limited amount of moisture at ambient condition^46^. Compared with the negligible uptake of water vapour, Fig. 3b shows that ZIF-8 possesses an S-shaped isotherm for methanol uptake demonstrating the framework affinity to methanol. Moreover, the trapped methanol molecules could reversibly escape from the cavities of ZIF-8 (yellow color area in Fig. 2b). The reversible and marginal water uptake endows ZIF-8 the merits of high immunity to humidity, and hence preserve the ZIF-8 based sensor from moisture attack, while cross-responding to humidity is regarded as one of most challenges for gas sensors. Moreover, it is possible to trap gases with large molecule size, e.g. toluene. This phenomenon should be largely attributed to the exothermal adsorption of toluene molecules (5.25 Å) from the relative small aperture of ZIF-8 (3.4 Å).

**Figure 4:**
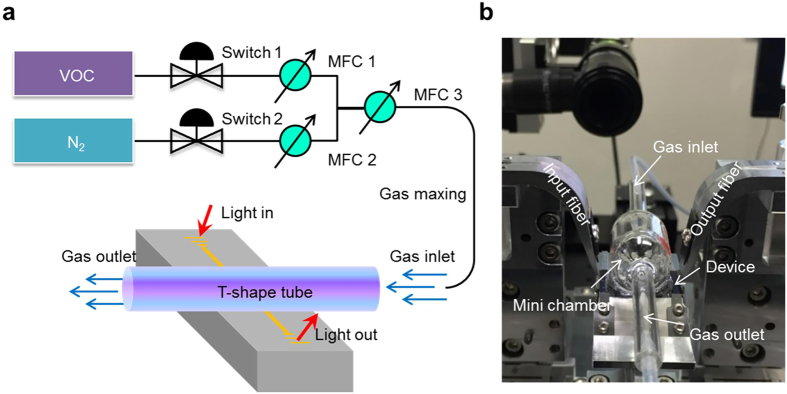
(**a**) Experimental apparatus using a gas mixture system and optical alignment system. (**b**) Illustration of the device sitting on the three-axis alignment system covered by a gas cell.

To measure the response of the photonic-MOF sensor in the presence of VOCs vapours, we utilized a gas mixing system and controlled the concentration by two mass flow controllers (MFCs) to control the carrier gas (N_2_) and the VOCs flow as shown in Fig. 4a. The gas flows were maintained at 100 sccm during the whole experiments. A T-shape glass gas cell was used to control the gas environment atop the chip that was affixed by a UV-glue. The species and pressure of the gas above the chip were governed by the flexible tubing connected to the glass cell. A photograph of the gas cell attached to a chip is shown in Fig. 4b. The chip was mounted on a vertical alignment system for light coupling between fibers and chip. The input light was from a tunable laser light source (ANDO AQ4321D) while the output light was received by an embedded power meter synchronized with the tunable laser source. Both the input and output signals were transmitted to a computer for real-time monitoring; the transmission spectra were plotted subsequently.

**Figure 5:**
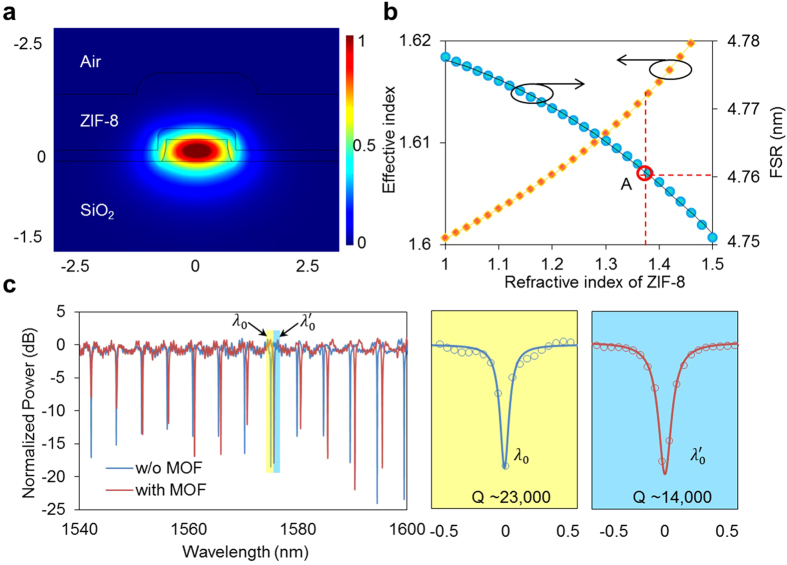
(**a**) Finite element method simulation results of the electrical component of the optical fields of the waveguide evanescently propagated in the hybrid photonic-MOF waveguide. (**b**) Theoretical calculation of the relationship between the refractive index of ZIF-8, effective index and FSR. (**c**) Transmission spectra measurements for resonator without MOF and with 1 μm thick MOF, and zoomed in view of the resonant dip.

The optical properties of the device were simulated and characterized. The optical fields of the MRR decaying exponentially outside its geometric boundaries were simulated by finite element method (FEM, Fig. 5a). The evanescent field, which has energy accounting for 5% of the total photonic energy, can sense the changes of ZIF-8’s RI effectively. In this simulation, we assume the dispersion of ZIF-8 at near infrared region is zero because of the small portion of the total light in ZIF-8 film^47^. The effective index generated by FEM also can be used to estimate the RI of ZIF-8 through calculating the free spectral range (FSR) of the MRR. From the FSR, we can identify the RI of the ZIF-8 and effective index of waveguide, as pointed by “A” as shown in Fig. 5b. Our calculation based on the thin layer of ZIF-8 with a thickness of 1 μm. Transmission spectra of the MRR were measured with (red line) and without (blue line) ZIF-8, as shown in Fig. 5c. The FSR of the MRR increasing from 4.65 nm to 4.76 nm was observed when 1-μm thick ZIF-8 was grown. The loaded Q-factor of the MRR was reduced from 23,000 to 14,000, as shown in the zoomed in view. The drop of the Q-factor induced by the growth of the ZIF-8 film was measured. It slightly increases the DL in the sensing due to the broaden 3-dB bandwidth. Based on the theoretical calculation of MRR, we can conclude that the ZIF-8 film has a refractive index of 1.38. This refractive index is on the lower end of the range of previously reported values, implying our film was more porous or had larger pores.

**Figure 6:**
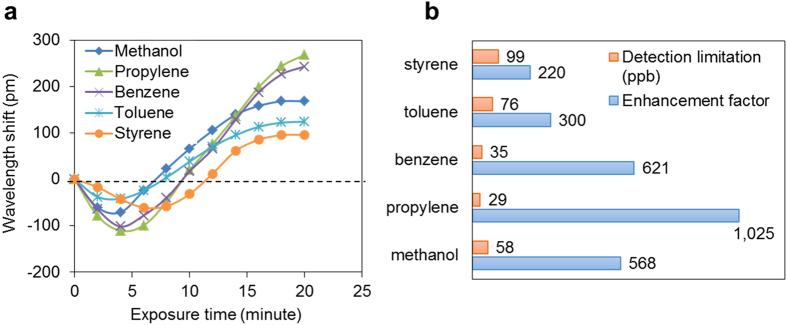
(**a**) Optical response of the hybrid photonic-MOF device to 20 minutes exposure of different VOCs with a fixed concentration of 100 ppm. (**b**) Enhancement factor and detection limitation of five VOCs at equilibrium.

As mentioned above, the resonant wavelength of the MRR is strongly dependent on the changes of ZIF-8 RI induced by gas adsorption. The wavelength shifts monitored at around 1575.67 nm are shown in Fig. 6a. Five VOC vapours: methanol, propylene, benzene, toluene, and styrene, were injected separately into the device with a fixed concentration of 100 ppm carried by N_2_ gas flows. The resonant wavelength exhibited a blueshift firstly, and then started to redshift for each gas, as shown in Fig. 6a. The blueshift happening at the initial stage is probably induced by air-cooling of the airflow and the heat exchange in the gas absorption. The redshift results from the increase in RI of ZIF-8 due to adsorption, which can offset the decrease of RI from the thermo-optic effect that can be well modelled with Fick’s equation of diffusion^48^,^49^. The equilibrium during the exposure time is between 30 to 45 minutes for these five VOC vapours, and the final shifts of the resonance are 172 pm, 341 pm, 282 pm, 131 pm, and 101 pm for methanol, propylene, benzene, toluene, and styrene, respectively. The long-time taken is attributed to the relative thick of the ZIF-8 film, as well as the unpassivated walls of the gas inlet tubing. In further studies, the long equilibrium time can be shortened by adopting thinner ZIF-8 films and advanced sensor packaging.

The sensitivity of the sensor can be defined as wavelength shift (Δλ) over gas concentration (C), (Δλ/C). The wavelength shift (Δλ) is determined by the refractive index changes variation (n − n_0_) with gas concentrations, which is expressed by initial resonant wavelength (λ_0_), the magnitude of evanescent field (t), and initial group index (n_g_).


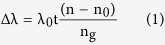


The refractive index of a gas mixture from the Lorenz-Lorentz equation is given by the relation^50^


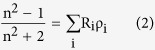


in which R_i_ and ρ_i_ are the specific refraction and the partial density of the i-type gas of the mixture, respectively. For 100-ppm VOCs mixture, the refractive index difference is as low as 10^−7^ RIU. It generates ~0.5 pm wavelength shifts at the best case if there is no pre-concentration, which is much smaller than the sensor with MOF coatings. Comparing to a MRR without pre-concentration/absorption layers, the hybrid photonic-MOF devices can improve the sensitivity up to 568, 1025, 621, 300, and 220 times for methanol, propylene, benzene, toluene, and styrene, respectively, as shown in Fig. 6b. Except the refractive index responding to different gases, the sensing performance also strongly relies on the uptake of VOCs molecule by ZIF-8 crystals^51^. For example, the low selectivity of styrene can be attributed to the fact that the styrene molecule (5.8 Å) is too bulky to penetrate into the cage of ZIF-8 because of the relative small aperture size of ZIF-8. As the resonant wavelength variation induced by the environmental temperature variation and tunable laser instability is around +/−0.1 pm in the experiments as previous mentioned^52^, the detection limits for these five VOC vapours are determined in the range from 29 to 99 ppb as shown in Fig. 6b. It is worth of noting that a more advanced temperature stabilization scheme and a higher accuracy wavelength tracker can be used for further improvement of detection accuracy.

The benchmark of the miniature MOF sensors is shown in Table 1. Based on the literature survey, there are three main solutions for gas detection by use of MOFs adsorption: (1) Mass weighting by use of coating MOFs onto Micro-electro-mechanical system (MEMS) resonators, in which the resonant frequency of the MEMS resonators changes as gas concentration^53^; (2) Electrical sensing via measuring electrical characters, e.g. resistivity and conductivity. When MOFs expose in different gas species or different gas concentration, MOFs’ electrical characters can be changed accordingly^54^; (3) Measuring optical properties of MOFs, for example the refractive index of MOFs changes as gases species and concentrations. As shown in the Table 1, the detection limitation of the 3^rd^ approach reported by this paper can be reduced further compared with weighting approaches and electrical sensing approaches. For the response time, it is strongly depended on the layer thickness of the MOF as discussed in previous section. Although the response time of 30 minutes is relative longer, it still can be adopted at some applications that require high-accuracy detection, such as indoor-air quality monitoring.

## Conclusions

The paper demonstrates the proof-of-concept of a versatile and highly integrated sensing platform for VOCs detection by utilizing appropriate MOF coatings to achieve selective functionalization. The CMOS-compatible photonic resonator is employed to determine the concentration of VOCs on-chip with a detection limitation of ppb-level. The presented platform can also be used as a flexible tool to deeply explore the optical properties of MOFs. Our results open an avenue towards the highly sensitive gas detection using MOF-coated devices.

## Additional Information

**How to cite this article**: Tao, J. *et al*. Hybrid Photonic Cavity with Metal-Organic Framework Coatings for the Ultra-Sensitive Detection of Volatile Organic Compounds with High Immunity to Humidity. *Sci. Rep.* **7**, 41640; doi: 10.1038/srep41640 (2017).

**Publisher's note:** Springer Nature remains neutral with regard to jurisdictional claims in published maps and institutional affiliations.

## Figures and Tables

**Table 1:** Compare of VOCs detection based on miniature sensor.

Solution	VOCs	Detection limitation	Response time	Reference
Microresonators with (poly)isobutylene coatings	Benzene	5.3 ppm	Not mentioned	53
	Toluene	1.2 ppm		
	*m*-Xylene	0.6 ppm		
Porous Co_3_O_4_ concave nanocubes	Ethanol	10 ppm	<10 s	54
	Acetone			
	Toluene			
	Benzene			
Hybrid photonic-MOF structure	Methanol	58 ppb	~30 minutes (depend on the MOF thickness)	This work
	Propylene	29 ppb		
	Benzene	35 ppb		
	Toluene	76 ppb		
	Styrene	99 ppb		
